# Effects of participation level and physical activity on eating behavior and disordered eating symptoms in the Brazilian version of the New Moves intervention: data from a cluster randomized controlled trial

**DOI:** 10.1590/1516-3180.2020.0420.R2.04022021

**Published:** 2021-05-10

**Authors:** Karin Louise Lenz Dunker, Marle dos Santos Alvarenga, Paula Costa Teixeira, Ruth Bartelli Grigolon

**Affiliations:** I PhD. Nutritionist and Professor, Department of Psychiatry, Universidade Federal de São Paulo (UNIFESP), São Paulo (SP), Brazil.; II PhD. Nutritionist and Professor, Department of Nutrition, Faculdade de Saúde Pública (FSP), Universidade de São Paulo (USP), São Paulo (SP), Brazil.; III PhD. Physical Educator and Professor, Institute of Psychiatry, Universidade de São Paulo (AMBULIM-IPq-HC-FMUSP), São Paulo (SP), Brazil.; IV MSc. Nutritionist and Professor, Department of Psychiatry, Universidade Federal de São Paulo, São Paulo (SP), Brazil.

**Keywords:** Obesity, Feeding and eating disorders, Adolescent behavior, Public health, Exercise, Eating disorders, Physical activity, Childhood and adolescence, Girls, Body image, Self-esteem

## Abstract

**BACKGROUND:**

Childhood and adolescent obesity is a worldwide public health concern. The New Moves program aims to change eating behavior (EB) and physical activity (PA).

**OBJECTIVE:**

To evaluate the effectiveness of an intervention and predictors of better outcomes relating to EB and PA levels.

**DESIGN AND SETTING:**

Secondary data from a cluster randomized controlled trial in 10 public schools in São Paulo, Brazil.

**METHODS:**

270 female adolescents, aged 12 to 14 years, were analyzed. Participation levels were categorized as presence in 1 to 9 sessions or 10 to 17 sessions, or control. Effectiveness was evaluated through improvement in disordered EB (DEB) and EB. Predictors of better outcomes relating to PA levels were evaluated through clustering of individual characteristics that affected changes in PA scores.

**RESULTS:**

Participation level was not significantly associated with changes in DEB or EB. Girls with higher body mass index percentile (BMI-P) percentile tended to have increases in sedentary lifestyles through the program. Girls with less body image dissatisfaction presented higher increases in daily PA. Girls with higher BMI-P percentile and higher self-esteem showed reductions in sedentary lifestyles. The program seemed to have more effect on daily PA among older girls than among younger girls.

**CONCLUSIONS:**

This program could be used as a structured action plan in schools, with the aims of improving eating behaviors and physical activity, in addition to promoting self-acceptance. The results indicate the importance of evaluating determinants of adherence, as these metrics might influence the effectiveness and future design of lifestyle programs.

## INTRODUCTION

Pediatric obesity is a worldwide health concern, and the majority of overweight or obese children live in low-to-middle income countries.^1^^,^^2^^,^^3^ Studies on low-income individuals^4^ and school-based interventions in low-to-middle income countries^5^^,^^6^ have demonstrated improvements in eating behavior (EB), physical activity (PA) and body weight. Traditional preventive measures against obesity that focus on weight seem to be ineffective and harmful to the participants. These programs contribute to eating and weight concerns, body image dissatisfaction, low self-esteem and unhealthy weight control practices. Such behaviors are considered to be risk factors for weight instability and development of eating disorders (ED).^7^^,^^8^

Obesity and ED result from cultural contexts that motivate an unhealthy relationship with food, EB and PA. In addition to these factors, this cultural context discourages respect for the diversity of body size.^9^ Obesity and ED share psychosocial and behavioral risk factors, which suggests that integrated interventions would lead to better outcomes.^9^ These integrated interventions can include overlapping of problems and involvement of similar risk factors (diet and weight). They make use of the economic efficiency of addressing two conditions in a single intervention.^10^

The New Moves program is an integrated intervention based on social cognitive theory, which is one of the most common theoretical frameworks used in interventions that have the aim of changing EB and PA.^11^^,^^12^^,^^13^ This program was designed in the United States focusing on adolescent females and it incorporates issues relating to eating disorders and obesity. It has a dynamic multi-component that includes factors that can predict body satisfaction, eating behaviors and patterns, weight control practices and PA levels. Positive outcomes were found among female adolescents in the United States, with improvements in sedentary lifestyles, eating patterns, unhealthy weight control behaviors and body/self-image.^11^

Considering that none of the previous intervention programs involved an after-school approach and that none of the studies with individuals from low socioeconomic backgrounds^5^^,^^6^ discussed the effect of participation level on the programs, a gap in the literature currently exists.

## OBJECTIVE

We aimed to evaluate the effectiveness of an intervention and the predictors of better outcomes relating to eating behaviors and PA levels.

We hypothesized that 1) girls with higher participation level would show significant improvements in those outcomes after the phase 1 (P1) intervention; and 2) their levels of body image dissatisfaction and self-esteem, their age and their nutritional status would enable prediction of PA levels and sedentary lifestyles.

## METHODS

### Study design

This study consisted of an exploratory analysis on a previously conducted cluster randomized controlled trial of the Brazilian New Moves program (BNMP). This program was conducted among girls aged 12 to 14 years old, at ten public schools in the central and southern areas of the city of São Paulo.^14^ The randomization of the original trial was performed at the school level to prevent contamination across students, between the intervention and observation arms. There was no blinding regarding intervention assignments or assessment, but blinding was present during data analysis. The analyses followed an intention-to-treat protocol that involved a sensitivity analysis in which all the subjects were included regardless of their length of follow-up or intervention. A total of 270 adolescent girls who practiced less than one hour of moderate to vigorous-intensity PA per day^15^ were randomized, among whom 131 were allocated to the intervention arm and 139 to the control arm. All the adolescents completed the baseline and endpoint assessments of P1 and the endpoint assessment of P2 (after nine weeks).

The BNMP consists of eight behavioral objectives that can be accessed at www.newmovesonline.com. However, in this study we focused on four behavioral objectives: (1) to be more physically active; (2) to increase fruit and vegetable intake; (3) to limit consumption of sugar-sweetened beverages; and (4) to eat breakfast every day. The BNMP was adapted for use as a nine-week intervention phase (P1) with 17 sessions that was followed, after a holiday interval, by an additional nine-week maintenance phase (P2) with 9 sessions, thus totaling 26 sessions. Adolescent girls were included from 2014 to 2015.

PA sessions were scheduled twice a week, and nutrition and social support components were held on those same days. All of these elements were conducted by trained healthcare professionals, including psychologists, dietitians and PA professionals.

The Institutional Review Board of the Federal University of São Paulo (Universidade Federal de São Paulo, UNIFESP), Brazil, approved our study on September 30, 2015 (CAAE: 06460112.6.0000.5505). Informed assent and consent forms were signed by the adolescents and their parents/guardians, respectively, before implementation of any study protocol. We registered the trial at the Brazilian Registry of Clinical Trials in September 2015 (RBR-6ddpb3).

### Participation level

The participation level was assessed by categorizing the number of times a participant was present in sessions promoted by the study intervention. This was considered in terms of three groups: control group, presence in 1 to 9 sessions and presence in 10 to 17 sessions. The exploratory analysis broke the randomization, so that participants in the intervention groups who did not participate in the intervention activities were analyzed as controls.

### Socioeconomic status (SES)

The “Brazilian Economic Classification” was used to categorize students based on their economic status. This classification is based on possession of items (e.g. television, radio or automobile) and the head of the family’s education level. From this, a score was generated and the participants were stratified according to monthly gross family income. The sum of these scores was used to determine the family’s purchasing power, which was categorized ranging from A1 to E.^16^ Based on Brazil’s monthly minimum wage (1 monthly minimum wage = US$ 249.50 at the time of writing this article, the categories are classified as A1/A2 = 11 minimum wages; B1 = 6 minimum wages; B2 = 3.12 minimum wages; C1 = 1.86 minimum wages; C2 = 1.27 minimum wages; and D/E = 0.89 minimum wages.

### Body mass index (BMI)

BMI was calculated as the weight in kilograms divided by the height in meters squared. Weight was measured by trained study staff using a digital scale, while height was measured with a portable stadiometer. Based on BMI-P percentiles (BMI-P) standardized according to age and sex, the participants were classified as defined by the World Health Organization (2007),^17^ as underweight or at risk (BMI-P ≤ 15), normal weight (15 < BMI-P < 85), overweight (85 ≤ BMI-P < 97) or obese (BMI-P ≥ 97).

### Disordered eating behaviors (DEB)

Disordered eating behaviors (DEB) were assessed using the following three scales.

Body Shape Questionnaire (BSQ) - Brazilian Portuguese version:^18^ This is a self-reported scale with the aim of assessing body image dissatisfaction. It presents good internal consistency (Cronbach’s alpha = 0.96) and reliability (r = 0.91; P < 0.001). The questionnaire consists of a 34-item self-reported scale that uses six Likert categories going from “never” to “always”, in which the higher the score is, the greater the degree of dissatisfaction with body image is. The scores were classified as “no dissatisfaction”, “slight dissatisfaction”, “moderate dissatisfaction” and “serious dissatisfaction”.^19^

Weight Control Behaviors Scale (WCBS) - Brazilian Portuguese version:^20^ This scale is an internally reliable and valid instrument that is used to assess factors representing healthy and unhealthy weight control behaviors. Out of the items on this scale, we used only the following nine items that assessed unhealthy weight control behaviors (UWCB): fasting, skipping meals, dieting, taking diet pills, making oneself vomit, using diuretics, using laxatives, using food substitutes like powdered/special drinks and smoking cigarettes. For each item, the participants responded with a “yes” or “no” response to indicate whether they had performed the behavior with the intent of losing weight in the past month. The internal consistency among these nine items was adequate (Cronbach’s alpha = 0.66). Through this scale, we aimed to assess unhealthy weight-control behaviors.

Rosenberg Self-Esteem Scale (RSES):^21^ This is a 10-item self-administered scale with items used as four-point Likert categories ranging from strongly agree to disagree, in which higher scores indicate higher self-esteem. Its construct validity shows a significant positive correlation with social support and its internal consistency has been reported as 0.68. With this scale, we aimed to assess self-worth and feelings about the self.

Although BSQ, WCBS and RSES are self-reported scales, we noticed in a pre-test study that the girls were having difficulties in filling out these questionnaires. We therefore trained the research staff to ask questions, without giving any interpretation of these questions.

### Eating behaviors (EB)

To assess EB, we evaluated changes in fruit, vegetable and sugar-sweetened beverage intake, and the frequency of breakfast intake. Fruit and vegetable intake was assessed using the following questions, “Thinking back over the past week, how many servings of fruit did you usually eat on a typical day?” and “Thinking back over the past week, how many servings of vegetables did you usually eat on a typical day?” The response options for both questions went from “none,” to “five or more servings/day.” Last week’s intake of regular soda and artificial juices was assessed using the following response options, which were converted to a mean intake/day: never = 0; once or twice a week = 0.2; three or four times a week = 0.5; once a day = 1; twice a day = 2; three times a day = 3; four times/day = 4; or five or more times/day = 5. The question relating to breakfast intake was, “During the past week, on how many days did you eat breakfast?” and the response options went from “none” to “seven days”.

### Physical activity level

The participants were provided with accelerometer devices (model GT3x, version 4.4.0; ActiGraph, Pensacola, FL, United States). They were instructed to fasten the elastic tape to their hips while keeping the instrument on their right side, to use it for seven consecutive days and only to remove it while showering. Data extraction and validation were conducted using the Actilife software, version 6.11.3 (ActiGraph, Pensacola, FL, United States).

Metabolic equivalents (METs) were evaluated as the rate of energy expenditure at rest. The accelerometer-related measurements of PA data corresponded to an index that was composed of a two-factor structure: daily PA (DPA) (Cronbach’s alpha = 0.94) and sedentary activity (Cronbach’s alpha = 0.79). Both structures have good factor loadings, internal reliability scores and appropriate validity across a range of self-reported PA measurements.^22^

The DPA index included the total time spent on moderate to vigorous PA (MVPA), total step count, Freedson bouts, METs and total time spent on mild-intensity PA. The sedentary activity index referred to sedentary lifestyles and included the following: total number of sedentary breaks, number of sedentary bouts, duration of sedentary activities and time spent doing vigorous and very vigorous PA.^22^

### Statistical analysis

Our exploratory analysis started with an evaluation of distributions, frequencies and percentages for each of the numerical and categorical variables of this study. The categorical variables were evaluated for near-zero variation,^23^ or categories with only a small percentage of response that could potentially bias our models. An extensive graphical exploratory analysis was used for both univariate analysis and bivariate associations between potential outcomes and the frequency of participation in study-related activities. Missing data were explored using a combination of graphical displays involving univariate, bivariate and multivariate methods. Imputation was performed using a k-nearest neighbors algorithm (n = 5).^24^

The association between participation level and outcome measurements was assessed using generalized estimating equations that adjusted for baseline variables, to account for each school level. In accounting for each school, we automatically controlled for the confounding of zero participation among those in the control group, compared with the intervention groups. We included both groups (control and intervention) while adjusting for their differences based on statistical considerations, thus providing greater statistical power for finding predictors of engagement, if they were present. The participation level was assessed by categorizing the count of the number of times a participant was present in the sessions, which was categorized into three groups. These groups were created to have approximately equal numbers of observations for each of the categories. The results were expressed as predicted means with 95% confidence intervals and were deemed statistically significant when the confidence intervals did not overlap between different estimates.

We also used regression trees (recursive partitioning) to evaluate how the clustering of individual characteristics affected the change in PA scores over time. The predictors included age, BMI-P, BSQ score, METs and RSES scale. The outcomes included the DPA and sedentary lifestyles. Regression trees represent the best cutoff points for predictor values in the context of a given outcome after previous features have been taken into account. This method allowed us to evaluate variables not only in isolation but also as a sequence. To avoid overfitting, we applied a cost-complexity strategy using weakest-link pruning by successively collapsing the internal node that produces the smallest per-node increase in the cost complexity criterion. When overfitting was detected, those nodes were removed. Otherwise, they were left intact. We also provided a graphical representation of each model. All analyses were performed using the R statistical language.^25^

## RESULTS

Out of the overall sample (n = 270), 65.2% of all the subjects did not participate in any sessions, 15.2% participated in 1 to 9 sessions and 19.6% participated in 10 to 17 sessions. Table 1 shows the baseline characteristics of the participants in each group. Analysis of variance (ANOVA) and chi-square analyses were conducted to determine whether the groups differed in any of the variables with continuous or categorical data, respectively. At the baseline, these groups differed in only three variables: age, SES and breakfast habits. Regarding the demographics of age and SES, these potentially confounding variables were entered as covariates in the remaining analyses.

**Table 1.** Participant characteristics stratified according to participation level

Variable	Total (270)	Control^**^ (176)	1 to 9 sessions^**^ (41)	10 to 17 sessions^**^ (53)	P-value
Age	13.4 (± 0.64)	13.5 (± 0.64)	13.3 (± 0.66)	13.1 (± 0.54)	< 0.001
Weight	53.3 (± 12.2)	53.75 (± 12.6)	51.32 (± 11. 6)	53.45 (± 11.7)	0.497
Height	1.58 (± 0.06)	1.58 (± 0.07)	1.57 (± 0.06)	1.57 (± 0.06)	0.181
Body mass index percentile	21.35 (± 4.4)	21.348 (± 4.5)	20.8 (± 4.2)	21.6 (± 4.2)	0.626
Body mass index percentile category					
Underweight or at risk (< P15)	26 (9.6%)	17 (9.7%)	5 (12.2%)	4 (7.6%)	0.978
Normal weight (P15-P85)	151 (55.9%)	100 (56.8%)	22 (53.7%)	29 (54.7%)	
Overweight (P85-P97)	59 (21.9%)	37 (21%)	9 (22%)	13 (24.5%)	
Obese (> P97)	34 (12.6%)	22 (12.5%)	5 (12.2%)	7 (13.2%)	
Socioeconomic status					
A2	7 (2.6%)	6 (3.4%)	0 (0%)	1 (1.9%)	0.004
B1	23 (8.5%)	14 (8%)	2 (4.9%)	7 (13.2%)	
B2	76 (28.1%)	61 (34.7%)	10 (24.4%)	5 (9.4%)	
C1	104 (38.5%)	56 (31.8%)	20 (48.8%)	28 (52.8%)	
C2	54 (20%)	38 (21.6%)	7 (17.1%)	9 (17%)	
D	6 (2.2%)	1 (0.6%)	2 (4.9%)	3 (5.7%)	
Rosenberg Self Esteem Scale					
Low self-esteem	8 (2.96%)	6 (3.43%)	2 (4.88%)	0 (0%)	0.322
Medium/high self-esteem	261 (96.7%)	169 (96.6%)	39 (95.1%)	53 (100%)	
Body Shape Questionnaire					
No dissatisfaction	167 (61.9%)	117 (66.5%)	23 (56.1%)	27 (50.9%)	0.11
Slight dissatisfaction	52 (19.3%)	26 (14.8%)	13 (31.7%)	13 (24.5%)	
Moderate/serious dissatisfaction	51 (18.9%)	33 (18.7%)	5 (12.2%)	13 (24.5%)	
Unhealthy Weight-Control Behaviors^*^					
No unhealthy weight-control behavior	190 (70.4%)	117 (66.5%)	31 (75.6%)	42 (79.2%)	0.261
1-3 unhealthy weight-control behaviors	73 (27.1%)	55 (31.2%)	8 (19.5%)	10 (18.9%)	
More than 5 unhealthy weight-control behaviors	7 (2.6 %)	4 (2.3%)	2 (4.9%)	1 (1.9%)	
Binge eating	39 (14.4%)	21 (7.8%)	10 (3.7%)	8 (2.9%)	0.127
Last week - fruits per day					
None	48 (17.8%)	31 (17.6%)	11 (26.8%)	6 (11.3%)	0.739
1 portion	65 (24.1%)	44 (25%)	10 (24.4%)	11 (20.8%)	
2 portions	84 (31.1%)	54 (30.7%)	10 (24.4%)	20 (37.7%)	
3 portions	41 (15.2%)	26 (14.8%)	6 (14.6%)	9 (17%)	
More than 3 portions	32 (11.9%)	21 (11.9%)	4 (9.8%)	7 (13.2%)	
Last week - vegetables per day					
None	71 (26.3%)	44 (25%)	16 (39%)	11 (20.8%)	0.223
1 portion	76 (28.1%)	48 (27.3%)	12 (29.3%)	16 (30.2%)	
2 portions	64 (23.7%)	40 (22.7%)	8 (19.5%)	16 (30.2%)	
3 portions	34 (12.6%)	25 (14.2%)	5 (12.2%)	4 (7.5%)	
More than 3 portions	25 (9.3%)	19 (10.8%)	0 (0%)	6 (11.3%)	
Last week - breakfast (number of days)	3.87 (± 2.7)	4.51 (± 2.59)	2.15 (± 2.49)	3.06 (± 2.46)	< 0.001
Last week frequency of regular soda/day	0.86 (± 1.3)	0.86 (± 1.32)	0.99 (± 1.36)	0.77 (± 1.18)	0.714
Last week frequency of artificial juice/day	1.23 (± 1.51)	1.14 (± 1.4)	1.16 (± 1.48)	1.6 (± 1.83)	0.242

Results are demonstrated as mean ± standard deviation, or crude number and percentage.

^*^Number of unhealthy weight control methods used by each participant, including fasting, skipping meals, dieting, vomiting, along with use of diet medications, diuretics, laxatives, nutritional replacements or cigarettes.

^**^Control group: participants who were absent from the program interventions; 1 to 9 sessions: participants who were present in 1 to 9 sessions of the program; 10 to 17 sessions: participants who were present in 10 to 17 sessions of the program.

To evaluate the association between the participation level and the outcome measurements, we used the three categories representing the participation level by means of a generalized estimating equation to account for the clustering effect within each school. The participation level did not result in statistically significant differences concerning outcome measurements, including body image dissatisfaction, self-esteem, unhealthy weight-control behaviors, BMI-P and EB measurements (frequency of breakfast and fruit, soda and artificial juice intake per day) (Table 2).

**Table 2.** Association between frequency of sessions and predicted means of outcome measurements (n = 270)

Outcome measurements	Control^**^ (n = 176)	1 to 9 sessions^**^ (n = 41)	10 to 17 sessions^**^ (n = 53)
**Body mass index percentile**	0.31 (0.11, 0.5)	0.44 (0.1, 0.78)	0.41 (0.09, 0.74)
**Body shape questionnaire**	-4.7 (-10.52, 1.07)	-6.8 (-15.21, 1.57)	-9.43 (-17.9, -0.99)
**Rosenberg Self Esteem Scale**	**0.93 (0.42, 1.44)**	**1.05 (0.27, 1.84)**	**0.8 (0.07, 1.53)**
Unhealthy weight-control behaviors^*^	0.004 (-0.15, 0.16)	-0.28 (-0.52, -0.05)	-0.23 (-0.44, -0.03)
**Last week frequency**			
Breakfast	-0.07 (-0.38, 0.25)	-0.41 (-0.96, 0.13)	-0.08 (-0.54, 0.38)
Fruits per day	-0.27 (-0.45, -0.09)	0.03 (-0.3, 0.36)	0.1 (-0.17, 0.37)
Vegetables per day	-0.05 (-0.23, 0.14)	-0.2 (-0.49, 0.1)	-0.16 (-0.44, 0.11)
Regular soda	-0.18 (-0.42, 0.06)	-0.05 (-0.41, 0.31)	-0.20 (-0.56, 0.15)
Artificial juice	0.24 (-0.07, 0.55)	0.09 (-0.33, 0.52)	-0.08 (-0.55, 0.39)

Results are demonstrated as predicted means with 95% confidence intervals.

^*^Number of unhealthy weight control methods used by each participant, including fasting, skipping meals, dieting, vomiting, along with use of diet medications, diuretics, laxatives, nutritional replacements, or cigarettes.

^**^Control group: participants who were absent from the program interventions; 1 to 9 sessions: participants who were present in 1 to 9 sessions of the program; 10 to 17 sessions: participants who were present in 10 to 17 sessions of the program.

Based on a previously validated sensor-based index,^22^ we used generalized estimating equations to identify predictors of change in daily PA and sedentary lifestyles between the baseline and 17 weeks. All the scores were normalized to a 0-100 scale, which was then used to determine the predicted means for the change in daily PA and sedentary lifestyles. Out of the 270 participants in the trial, 75 were excluded because of lack of data recording devices, or because they missed the orientation and training day when the devices were distributed, or because some data was found to be missing at the time of data extraction from the device. The analysis was conducted with a final sample of 195 participants, given that only these girls presented MET data. We found that the differences in participation level relating to 1 to 9 and 10 to 17 sessions gave rise to statistically significant associations with increases in sedentary lifestyle (P = 0.027 and P = 0.039, respectively) (Table 3).

**Table 3.** Changes in daily physical activity and sedentary activity (n = 195)

Participation level	Daily physical activity	P-value	Sedentary activity	P-value
Control^*^	2.83 (-3.99, 9.66)		-3.89 (-12.3, 4.53)	
1 to 9 sessions^*^	7.32 (-0.174, 14.8)	0.435	12.1 (4.11, 20.1)	0.027
10 to 17 sessions^*^	3.8 (-4.67, 12.3)	0.885	13 (2.1, 23.9)	0.039

Results are demonstrated as predicted means with 95% confidence intervals.

^*^Control group: participants who were absent from the program interventions; 1 to 9 sessions: participants who were present in 1 to 9 sessions of the program; 10 to 17 sessions: participants who were present in 10 to 17 sessions of the program.

**Figure 1.**
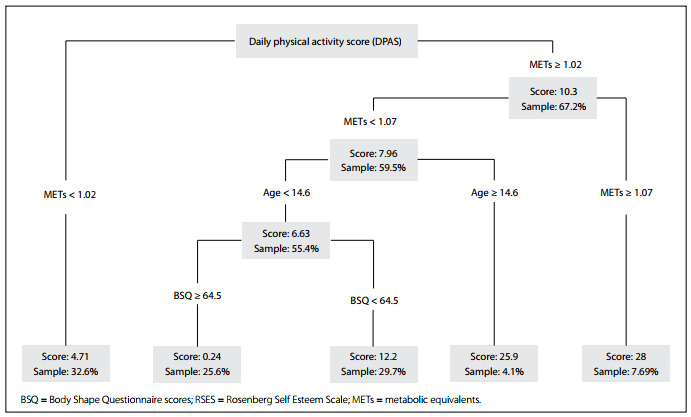
Regression tree of daily physical activity score changes (n = 195).

To better understand how the clustering of individual characteristics might affect their change in daily PA scores (DPAS) over time, we used regression tree analysis (recursive partitioning). We found that METs ≥1.02 was the first node separating those with higher or lower changes in DPAS. Individuals with lower METs showed a reduction in DPA, while individuals with higher METs showed an increase in DPA. At a METs cutoff point of 1.07, individuals with higher METs showed an increase in DPA, while individuals with lower METs showed a decrease in DPA. The second node in the tree was age. Accordingly, participants with METs between 1.02 and < 1.07 and age ≥14.6 years old demonstrated an increase in DPA, while participants with the same METs but who were younger than 14.6 years old showed a decrease in DPA. The third node in the tree was body image dissatisfaction (BSQ), which was associated with girls younger than 14.6 years old and METs between 1.02 and < 1.07. The cutoff point for BSQ was 64.5. Girls with body image dissatisfaction showed small changes in DPA, while those with less body image dissatisfaction showed an increase in DPA (Figure 1).

**Figure 2.**
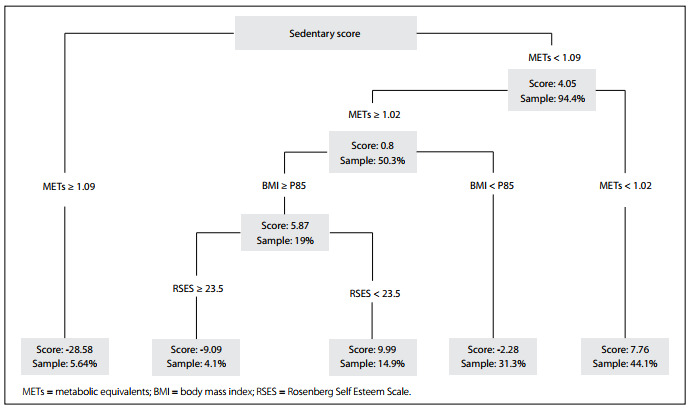
Regression tree of sedentary score changes (n = 195).

In evaluating predictors of changes in sedentary lifestyles (sedentary score, SS), METs were the first node in the regression tree. Girls with higher METs showed a reduction in SS, while those with lower METs showed an increase in SS. Also, girls with lower METs (< 1.09) showed an increase in the SS, while those with higher METs only showed a subtle increase in SS. The second node in the tree was BMI-P. Girls with lower METs and BMI-P less than P85 showed a reduction in SS, compared with those with BMI-P ≥P85, who showed an increase in SS. Girls with BMI-P ≥P85 and higher RSES showed a reduction in SS, while those with lower RSES showed an increase in SS (Figure 2).

## DISCUSSION

Our study demonstrated that the participation level in the BNMP did not affect DEB and EB. Also, we observed a negative effect on PA levels (an increase in sedentary lifestyles). In brief, girls with less body image dissatisfaction presented higher increases in daily PA, while for those with more body image dissatisfaction, the program had almost no effect on daily PA. The program seemed to have more effect on daily PA among older girls than among younger girls. Therefore, girls with higher BMI-P tended to have increasingly sedentary lifestyles, while eutrophic girls tended to reduce sedentary lifestyles. Those with higher BMI-P and higher self-esteem showed reductions in sedentary lifestyles, while those with higher BMI-P and lower self-esteem showed increases in sedentary lifestyles.

For the program to be successful, participation by everyone involved in the environment of these girls is necessary. Part of the program consists of communication with parents, so that they may motivate teenagers to improve their PA lifestyle. During the intervention, absence of parent and teacher involvement was observed. There was no motivation for PA at school, even though it formed part of the students’ curriculum. Souza et al.^26^ demonstrated that, both for children and for adolescents, the main adherence factors for PA were motivation and support from friends or parents, pleasure in doing PA and the opportunity to be with and play with other children and adolescents. Ceschini et al.^27^ and Moraes et al.^28^ demonstrated high prevalence of physical inactivity among adolescents, and the factors associated with this were gender (girls were more inactive than boys), age, lower socioeconomic status, geographical region of the city of São Paulo, daily time spent watching television and being obese.

One potential protective factor against body dissatisfaction is greater amounts of PA, given that this has been correlated with satisfaction with body image, self-esteem, reduced risk of ED and psychological benefits.^29^ In our sample, being an older girl, with less dissatisfaction with body image, normal BMI-P and better self-esteem was predictive of higher PA levels and less sedentary behavior. Girls with more body satisfaction may be more engaged in PA for reasons of fun and socialization, while girls who are dissatisfied with their bodies aim to change their weight and shape. For older girls, there was better understanding of healthcare, while younger girls were more motivated to play than to practice PA. Girls with higher BMI percentiles had more sedentary habits than did eutrophic girls, and this was a possible explanation for being overweight. Girls with higher self-esteem tended to be better motivated to exercise than did those with low self-esteem. Moreover, PA interventions were associated with increased self-concept and self-worth among children and adolescents.^30^

No improvement in EB was observed, given that socioeconomic issues and food availability may influence such habits. One possible explanation for this is that marketing of food and beverages may influence children’s food choices, preferences and consumption, especially with regard to foods that are high in energy density and poor in micronutrients.^31^ Another possible explanation is that manufactured products, with low monetary value and low dietary value, are preferred over healthy foods.^32^ Data from the latest 2008/2009 Brazil Household Budget Survey found that low-income communities tend to buy fewer fruits, vegetables, tubers and roots in a week than do the middle and upper classes. This may be consequent to a lack of food and nutrition education in public schools and for the overall community, which would develop skills and knowledge and allow these communities to choose and consume their foods safely and appropriately.^33^

Effective procedures and designs for programs targeting multicomponent behaviors among low-income individuals^4^ are still a challenge because these are not maintained over the long term. Certain limitations can influence the effectiveness of such programs.^4^^,^^34^^,^^35^ These limitations have been described as insufficient knowledge and understanding within the school community (participants, teachers, etc.) about the importance of healthy lifestyles.^36^

The strengths of our study are its use of reliable measurements for DEB and accelerometer devices for measuring PA levels. Nonetheless, our results should be interpreted in the light of some limitations of this study: 1) the sample was composed of adolescent girls, thus preventing generalization to adolescent boys; 2) the participants’ education levels and ages are likely to have been an interpersonal barrier to innovative intervention; 3) Twenty-eight percent of the girls who were initially randomized to the intervention arm completed all the assessments but did not participate of the sessions, which thus represents low adherence and will have impacted on the effectiveness of the program.

## CONCLUSIONS

This program could be used as a structured action plan in schools, with the aims of improving eating behaviors and physical activity, in addition to promoting self-acceptance. Moreover, it could be implemented in a personalized and interactive manner, with weekly messages on mobile devices. As discussed by López-Guimerà et al.,^37^ in interactive programs like New Moves, it is also important to monitor adherence to activities.

With regard to future work, researchers are encouraged to address the issue of adherence in after-school programs and to involve school staff in the development of further interventions. Moreover, through adding qualitative data to evaluations, insights that would enhance the likelihood of successful intervention may be gained.
